# How do childhood abuse and neglect affect prosocial behavior? The mediating roles of different empathic components

**DOI:** 10.3389/fpsyg.2022.1051258

**Published:** 2023-01-17

**Authors:** Peiyi Chen, Qiaofen Zhang, Xiyuan Sun, Xiaoyang Ye, You Wang, Xueling Yang

**Affiliations:** ^1^Department of Psychology, School of Public Health, Southern Medical University, Guangzhou, Guangdong, China; ^2^Department of Artificial Intelligence, Guilin University of Electronic Technology, Guilin, Guangxi, China; ^3^Department of Psychiatry, Zhujiang Hospital, Southern Medical University, Guangzhou, Guangdong, China

**Keywords:** abuse, neglect, prosocial behavior, perspective-taking, empathic concern, personal distress

## Abstract

**Background:**

Childhood abuse and neglect are typically considered as two different forms of maltreatment. Previous international studies have found differential effects of abuse and neglect on prosocial behavior, but this and the mediating pathway underlying these associations have not been examined in a Chinese sample. Our study aims to examine the effects of childhood abuse and neglect on prosocial behavior in Chinese participants and test the unique mediating roles of different empathic components in these associations.

**Methods:**

A total of 1,569 young adults (average age = 18.17 years) were recruited from a college that enrolls students from all provinces of China. Participants completed a series of questionnaires, including the Childhood Trauma Questionnaire, Interpersonal Reactivity Index, and Prosocial Tendencies Measure. Path analysis was conducted to determine the mediational relationships.

**Results:**

Emotional neglect had significant direct effect on prosocial behavior (*β* = −0.108, *p* < 0.001), and could also impact prosocial behavior through the mediating roles of perspective-taking and empathic concern (effect size = −0.091 and −0.097 respectively, *p* < 0.001). Emotional abuse affected prosocial behavior only through personal distress (effect size = −0.072, *p* < 0.001). Physical abuse, sexual abuse and physical neglect have little effect on prosocial behavior and empathy.

**Conclusion:**

Childhood abuse and neglect have distinct influences on prosocial behavior. Emotional abuse and emotional neglect affect prosocial behavior through distinct pathways. This conclusion could help to establish precise interventions for improving prosocial behavior in maltreated individuals.

## Introduction

Prosocial behavior refers to a broad range of actions that are intended to benefit other people or an ongoing political system, including helping, donating, comforting, sharing and volunteering (Dovidio, 2001; Penner et al., 2005). Living in a complex social environment, it is almost impossible for humans to avoid social contact and social reciprocity. Previous studies have demonstrated that positive social interaction, such as prosocial behavior, can effectively improve people’s emotional distress, enhance well-being (Lin et al., 2019), decrease internalizing and externalizing problems (Memmott-Elison et al., 2020), and decrease morbidity of mental diseases (Schacter and Margolin, 2019). In particular, young adults’ prosocial behavior can help them develop social skills, establish solid social relationships, and engage in society (Hu et al., 2019; Memmott-Elison et al., 2020). Considering the positive effects of prosocial behavior on individuals’ lives, it is necessary to investigate the related factors of and psychological mechanisms underlying prosocial behavior.

### Childhood abuse/neglect and prosocial behavior

According to learning theories, individuals develop prosocial belief, internalize moral standard and acquire helping skills through interactions with their parents or other caregivers (Schuhmacher et al., 2019). Therefore, abnormal parent–child relationships or unhealthy growth environments, such as childhood maltreatment, might hinder the development of prosocial behavior in later life (Music, 2011; Carvalho et al., 2020; Wu et al., 2020; Prior et al., 2021).

Childhood maltreatment, including abuse (emotional, physical, and sexual abuse) and neglect (emotional and physical neglect), are relatively common around the world, with prevalence rates ranging from 41% to 97% (Carlson et al., 2020). It is widely acknowledged that childhood abuse and neglect should be considered as two different forms of maltreatment. According to the conceptual framework of McLaughlin et al. (2014a), abuse should be categorized as experiences of threat, and neglect as experiences of deprivation. Similarly, Humphreys and Zeanah (2015) argued that both childhood abuse and neglect are deviations from the expectable environment, but in different directions (abuse as the presence of harmful input, while neglect as a lack of necessary input), and suggested that different risks for psychopathology and later-life outcomes emerge from these two types of abnormal environmental input. Several studies have examined the distinctions between abuse and neglect in the context of psychiatric disorders (Vonderlin et al., 2018; Cohen and Thakur, 2021; Villodas et al., 2021), substance abuse disorder (Kobulsky et al., 2018), accelerated aging (Colich et al., 2020) and brain structure and cognitive function (Teicher and Samson, 2013, 2016; McLaughlin et al., 2014b; Kim-Spoon et al., 2021). Based on the above theoretical frameworks and empirical studies, some scholars have suggested that it is no longer suitable to use a cumulative risk approach (that only considers the number and severity of traumatic exposures, and/or which simplifies or ignores the distinct effects of different forms of maltreatment) to assess the unique mechanisms linking particular maltreatments with developmental outcomes (Teicher and Samson, 2016). Therefore, we independently examined the effects of abuse and neglect on prosocial behavior in the current study.

To date, only three studies have investigated the different associations of abuse and neglect with altruistic attitudes (a type of prosocial behavior) among young adults (Carvalho et al., 2020; Gomis-Pomares and Villanueva, 2020; Prior et al., 2021). Two studies in European found that, after controlling for other types of maltreatment, only emotional neglect and physical abuse significantly predicted a low level global altruistic attitudes and behavioral expressions of altruism (Carvalho et al., 2020; Gomis-Pomares and Villanueva, 2020). Another study conducted in Australia found that only physical neglect was negatively associated with affective altruism, after controlling for demographic variables (Prior et al., 2021). These studies suggest that childhood maltreatment, especially neglect, hinders prosocial behavior. Additionally, they also showed that differences in emotional and physical maltreatment on prosocial behavior exist, which suggests that further distinction between abused or neglected expriences is necessary (for example, divide abuse into emotional, physical and sexual abuse, and divide neglect into emotional and physical neglect).

### The mediating role of empathy

Abuse and neglect that occurs in childhood or early adolescence are considered as a distal influencing factor of adulthood prosocial behavior. Thus, we predicted that childhood abuse and neglect affect prosocial behavior through more proximal traits or tendencies. In the present study, we considered the potential mediating role of empathy.

Empathy broadly refers to the multidimensional ability to understand others’ cognitive states and share others’ emotions (Eisenberg and Miller, 1987). Empathy is crucial for developing prosocial behavior (Eisenberg et al., 2006; Carrizales et al., 2021). Many scholars have suggested that empathy should be divided into cognitive and emotional components (Davis, 1983). The Interpersonal Reactivity Index (IRI) is a well-established assessment tool for different empathic components, in which cognitive empathy includes perspective-taking (PT) and fantasy (FS) components. PT, also called theory of mind (ToM), is defined as the ability to adopt others’ psychological perspective and reason their views, thoughts and emotions (Davis, 1983; Decety, 2011). In behavioral studies, the competence and accuracy of emotion recognition are the important embodiments of PT. FS refers to the tendency to imagine oneself as fictitious characters. Emotional empathy refers to the capacity to sense and share others’ feelings, including empathic concern (EC) and personal distress (PD; Davis, 1983; Shamay-Tsoory, 2011; Guhn et al., 2020). EC is defined as the other-oriented empathic tendency, and is characterized by the feelings of warmth, compassion, and concern for needy people. PD has been described as the self-oriented discomfort in response to other people’s situations or conditions, such as anxiety, distress, and unease.

The empathy-altruism hypothesis argues that empathy can evoke altruistic motivation which elicits more prosocial behavior in the future (Batson, 1987), but that not all the components of empathy benefit prosocial behavior. Previous theories and studies have suggested that there are differential impacts of different empathic components on prosocial behavior. For example, EC and PT have been reported to be positively associated with prosocial behavior (Carlo et al., 2015; Bowman-Smith et al., 2021), while PD has been reported to be unrelated to or negatively associated with prosocial behavior (Eisenberg et al., 1989; Batson and Shaw, 1991). Moreover, there has been mixed evidence on how FS impacts prosocial behavior. For example, FS has been found to elicit prosocial behavior in young adults (Tahiroglu and Taylor, 2019), but another study found that FS had little influence on prosocial behavior after controlling for confounders (Pang et al., 2022). Further examining the relationships between different components of empathy and prosocial behavior in a broader population may help to clarify the inconsistencies of prior studies.

Empathy can be damaged by childhood maltreatment (Prino and Peyrot, 1994; Levy et al., 2019). Previous research has indicated that empathy emerges in early life and develops for a long period after that through abundant interactions with caregivers (De Haan and Gunnar, 2009). The parent–child attachment bond provides a template for children to understand and resonate with the pain, feelings, and thoughts of others (Feldman, 2017). However, being abused or neglected by caregivers in early life, could disrupt the normal development of empathy. Empirical studies have demonstrated that more severe childhood maltreatment predicted lower emotional and cognitive empathy (Locher et al., 2014; Mielke et al., 2016). As mentioned, the different features of abuse and neglect may mean that they have differential impacts on empathy and its components. Neglected children, who lack emotional cue input in early life, might have more damage in empathic development than abused children, who have sufficient but harmful cue input. One study found that both emotional and physical neglect, but not abuse, predicted lower empathy, as partially suggested by the above hypothesis (Ometto et al., 2016). However, to our knowledge, few studies have investigated the effects of separate forms of maltreatment on empathic components, and the majority of these studies only focused on childhood abuse (Perez-Albeniz and de Paul, 2004; Mielke et al., 2016; Meidan and Uzefovsky, 2020). Considering that abuse and neglect usually occur together, it is difficult to accurately assess the effect of one maltreatment form on empathy without controlling for the other. Based on these previous findings and limitations, it seems necessary to investigate the associations between the different forms of maltreatment and empathic components more extensively. More importantly, it is still not well understood how these associations are linked with prosocial behavior. One well-established study found that lower general empathy mediated the association between childhood maltreatment and reduced prosocial behavior (Yu et al., 2020), which suggests that empathy is a promising mediator, and more research is needed to explore the mediating effects of different forms of empathy. Based on above studies, we further proposed hypotheses: Firstly, neglect have more profound effects on both empathy and prosocial behavior than abuse. At the same time, emotional maltreatment have more significant influences on empathy and prosocial behavior than other forms of maltreatment. Secondly, abuse and neglect could impact prosocial behavior differently *via* distinct empathic responses.

### The current study

Reviewing the existing literature, little research has investigated the mediating pathway underlying the relationships among abuse, neglect, and prosocial behavior and scarce studies focused on the differential effects of abuse and neglect on different empathic components and prosocial behavior. Therefore, the current study conducted a cross-sectional investigation by using a sample of Chinese young adults to separately explore the unique influence of abuse or neglect on prosocial behavior, and examine the special roles played by different empathic components.

## Materials and methods

### Participants

The questionnaire survey was conducted among 1,652 college students (aged between 16 and 22 years) with cluster sampling method from Southern Medical University in Guangdong Province, which enrolls students from all provinces in China. All participants volunteered to complete an online questionnaire survey in the classroom. Questionnaires with more than 10% missing values were considered as invalid. Besides, we excluded the participants who reported that they have had been diagnosed as severe mental illnesses, such as schizophrenia, bipolar disorder and so on. After excluding these, the sample included a total of 1,569 participants (639 males and 930 females, average age of 18.17 years). Among the participants, 54.0% came from urban areas, and 22.1% were from a town, and 23.9% were from rural areas. A total of 473 participants (30.1%) were only children.

### Procedure

Before conducting the investigation, ethical approval was granted by the Ethics Committee of the first author’s college. All the survey data were collected after informed consent had been obtained from the participants. Participants were told that their personal information would be protected, and they were free to quit the survey at any time without any punishment. To enhance the validity of the responses, participants filled in the questionnaires anonymously.

### Measurement tools

#### Childhood abuse and childhood neglect

Childhood abuse and neglect were measured using the Chinese version of the Childhood Trauma Questionnaire-Short Form (Bernstein et al., 2003; Zhao et al., 2005), which is a widely used tool to assess the type and severity of childhood maltreatment. This 28-item self-reported scale contains the five following subscales: emotional abuse (EA), physical abuse (PA), sexual abuse (SA), emotional neglect (EN), and physical neglect (PN). Each subscale has five items that are scored on a five-point Likert scale that ranges from 1 (never) to 5 (always). In the present study, childhood abuse was divided into emotional abuse, physical abuse and sexual abuse, and childhood neglect was divided into emotional neglect and physical neglect. The total abuse score and total neglect score were calculated by summarizing the scores of related subscales. Higher scores indicated higher levels of maltreatment. Participants with scores for emotional abuse ≥13, physical abuse ≥10, or sexual abuse ≥8 were considered as having had “significant abuse experiences,” and those with scores for emotional neglect ≥14 or physical neglect ≥10 were considered as having had “significant neglect experiences” (Cheng et al., 2021). In this study, the Cronbach’s α coefficient of the Childhood Trauma Questionnaire-Short Form was 0.824, and the Cronbach’s α for the abuse subscales and neglect subscales were 0.704 and 0.816, respectively.

#### Empathy

Empathy was measured using the Chinese Version of the IRI (C-IRI), which is a 28-item self-report questionnaire. The C-IRI is a multidimensional measure to assess empathy (Davis, 1980, 1983), and comprises four subscales including EC, PT, PD, and FS. In the current study, we used these four subscales to assese different empathic component. The Cronbach’s α coefficients for the four subscales in this study ranged from 0.621 to 0.873.

#### Prosocial behavior

The Chinese version of Prosocial Tendencies Measure was used to assess prosocial behavior (Carlo and Randall, 2002; Kou et al., 2007). The Prosocial Tendencies Measure consists of 26 items that are rated on a five-point Likert scale ranging from 1 (does not describe me at all) to 5 (describes me greatly). The measure assesses six domains of prosocial behavior (emotional, public, anonymous, dire, altruism, and compliant). In this study, the Cronbach’s α coefficient of the Prosocial Tendencies Measure was 0.782, and the Cronbach’s α coefficients for the six subscales ranged from 0.576 to 0.805.

#### Data analysis

First, IBM^®^ SPSS 22.0 was used to obtain the descriptive statistics and examine correlations. A descriptive analysis was performed to summarize the sociodemographic, using the mean and standard deviations or the number and percentage distribution. Distribution of the main variables (including all forms of childhood maltreatment, prosocial behavior, four forms of empathy) are slightly skewed with the Skewness ranged from −0.17 to 1.53, and the Kurtosis ranged from −0.28 to 2.37. West et al. (1995) and Kim (2013) proposed that the data with an absolute skew value lower than 2 and an absolute kurtosis value lower than 7 could be considered as basically normal distribution. Besides, parametric test including Pearson’s correlation test, Structure Equation Modeling and *t*-test are robust even for skewness and nonnormality (Norman, 2010; Fagerland, 2012). Thus, we conducted Pearson’s correlation analysis to examine the correlations between main variables. We used Bonferronic correction to correct the statistical values of multiple testing (Armstrong, 2014). Second, based on the results of Pearson’s correlation, we performed path analysis to examine the mediating roles of different empathic components in the associations between childhood abuse or neglect and prosocial behavior using the Process macro software 4.1 (Preacher and Hayes, 2004) in SPSS. Firstly, we examined the effects of abuse or neglect on different empathic components. And then we examined the effects of abuse or neglect on prosocial behavior which included different empathic components. We used *R*^2^ and *F* value to present the explanatory powers and the significance level. Bootstrapping with 5,000 iterations was used to test the significance of direct and indirect effects. Age, sex and other sociodemographic variables were controlled for as covariates in the mediating analyses. What’s more, *t*-test was conducted to examine gender differences in childhood trauma, empathic components and prosocial behavior.

## Results

### Preliminary analyses

Descriptive statistics for the full sample are presented in Table 1. Abuse alone was experienced by 11.3% of participants (*n* = 177), 16.6% (*n* = 260) were exposed to neglect alone, and 8.2% (*n* = 129) were exposed to mixed childhood maltreatment. About 36.1% (*n* = 566) individuals reported at least one form of maltreatment in the current study. Correlations between the main variables are summarized in Table 2.

**Table 1:** Socio-demographic characteristics of the full sample (*N* = 1,569).

Variables	Categories	*N* (%)
Age (Mean ± SD)		18.17 (0.64)
Sex	Male	639 (40.7)
	Female	930 (59.3)
Hometown	Urban	848 (54.0)
	Town	346 (22.1)
	Rural	375 (23.9)
Siblings	None	473 (30.1)
	1	617 (39.3)
	2	285 (18.2)
	More than 2	194 (12.4)
Childhood maltreatment	Abuse	177 (11.3)
	Neglect	260 (16.6)
	Mixed	129 (8.2)
	At least one form of maltreatment	566 (36.1)

SD, standard deviation.

**Table 2:** Pearson’s correlations, means, and SDs of the main variables (*N* = 1,569).

	Abuse	EA	PA	SA	Neglect	EN	PN	EC	PT	PD	FS	PB
Abuse	1											
EA	0.859***	1										
PA	0.698***	0.333***	1									
SA	0.427***	0.164***	0.105**	1								
Neglect	0.419***	0.458***	0.217***	0.062	1							
EN	0.431***	0.473***	0.219***	0.066	0.946***	1						
PN	0.281***	0.303***	0.153***	0.039	0.816***	0.584***	1					
EC	−0.010	−0.014	−0.039	0.062*	−0.114***	−0.106**	−0.095*	1				
PT	−0.078	−0.071	−0.061	−0.012	−0.119***	−0.125***	−0.075	0.215***	1			
PD	0.164***	0.196***	0.076	−0.002	0.108**	0.113**	0.070	0.134***	−0.048	1		
FS	0.152***	0.168***	0.075	0.025	0.022	0.028	0.005	0.199***	0.082	0.200***	1	
PB	−0.063	−0.100*	0.015	−0.013	−0.179***	−0.185***	−0.117***	0.347***	0.344**	−0.079	0.092*	1
Mean ± SD	18.29 ± 3.48	7.15 ± 2.32	5.85 ± 1.56	5.29 ± 0.94	15.56 ± 5.23	8.98 ± 3.72	6.58 ± 2.10	23.89 ± 2.74	23.80 ± 2.72	23.00 ± 3.72	21.80 ± 2.72	94.23 ± 11.00

Corrected by Bonferronic Corretion. *p < 0.05; **p < 0.01; ***p < 0.001; Abuse, childhood abuse; Neglect, childhood neglect; EA, emotional abuse; PA, physical abuse; SA, sexual abuse; EN, emotional neglect; PN, physical neglect; EC, empathic concern according to the Interpersonal Reactivity Index (IRI); PT, perspective-taking according to the IRI; PD, personal distress according to the IRI; FS, fantasy according to the IRI; PB, prosocial behavior according to the Prosocial Tendencies Measure; SD, standard deviation.

**Figure 1:**
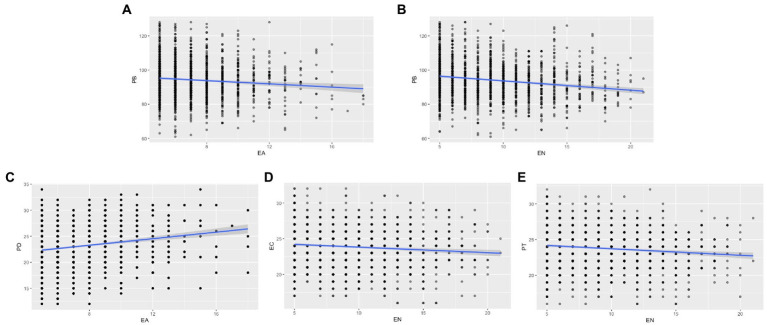
Scatterplots of the main correlations. EA, emotional abuse; EN, emotional neglect; PB, prosocial behavior; PD, personal distress; EC, empathic concern; PT, perspective-taking. 1Scatterplots of the main correlations. EA, emotional abuse; EN, emotional neglect; PB, prosocial behavior; PD, personal distress; EC, empathic concern; PT, perspective-taking. **(A)** correlation between EA and PB; **(B)** correlation between EN and PB; **(C)** correlation between EA and PD; **(D)** correlation between EN and EC; **(E)**, correlation between EN and PT.

Only emotional abuse, emotional neglect and physical neglect were significantly negatively correlated with prosocial behavior (*r* = −0.100, *p* < 0.05; *r* = −0.185, *p* < 0.001; *r* = −0.117, *p* < 0.001), while physical abuse and sexual abuse were not (*r* = 0.015, *r* = −0.013, *p* > 0.05). Emotional abuse was significantly correlated with PD, and FS (*r* = −0.196, 0.168, respectively; *p* < 0.001), but uncorrelated with EC and PT (*r* = −0.014, *r* = −0.071, respectively; *p* > 0.05). Physical abuse and sexual abuse were uncorrelated with any form of empathy. Emotional neglect was correlated with EC, PT, and PD (*r* = −0.106, *p* = 0.004; *r* = −0.125, *p* < 0.001; *r* = −0.113, *p* = 0.001), but uncorrelated with FS (*r* = 0.028, *p* > 0.05). Physical neglect was only correlated with EC (*r* = −0.095, *p* < 0.05). Detailed information were presented in Table 2. The scatterplots of significant correlations were present in Figure 1.

Besides, we found that males have more FS than females (*t* = 3.85, *p* < 0.001), while females have higher EC and PD than males (*t* = −3.25, *p* < 0.001; *t* = −8.23, *p* < 0.001). But there were no significant difference in abuse, neglect and prosocial behavior between males and females (*t* = −1.67, *p* = 0.094; *t* = −1.628, *p* = 0.104; *t* = −0.809, *p* = 0.419).

### Empathic concern and perspective-taking mediated the association between emotional neglect and prosocial behavior

Based on the results of Pearson’s correlation, we found both emotional and physical neglect were significantly associated with prosocial behavior and some empathic components. Therefore, we conducted mediating analysis to investigate the special roles of empathic components in the relationship between emotional or physical neglect and prosocial behavior.

**Figure 2:**
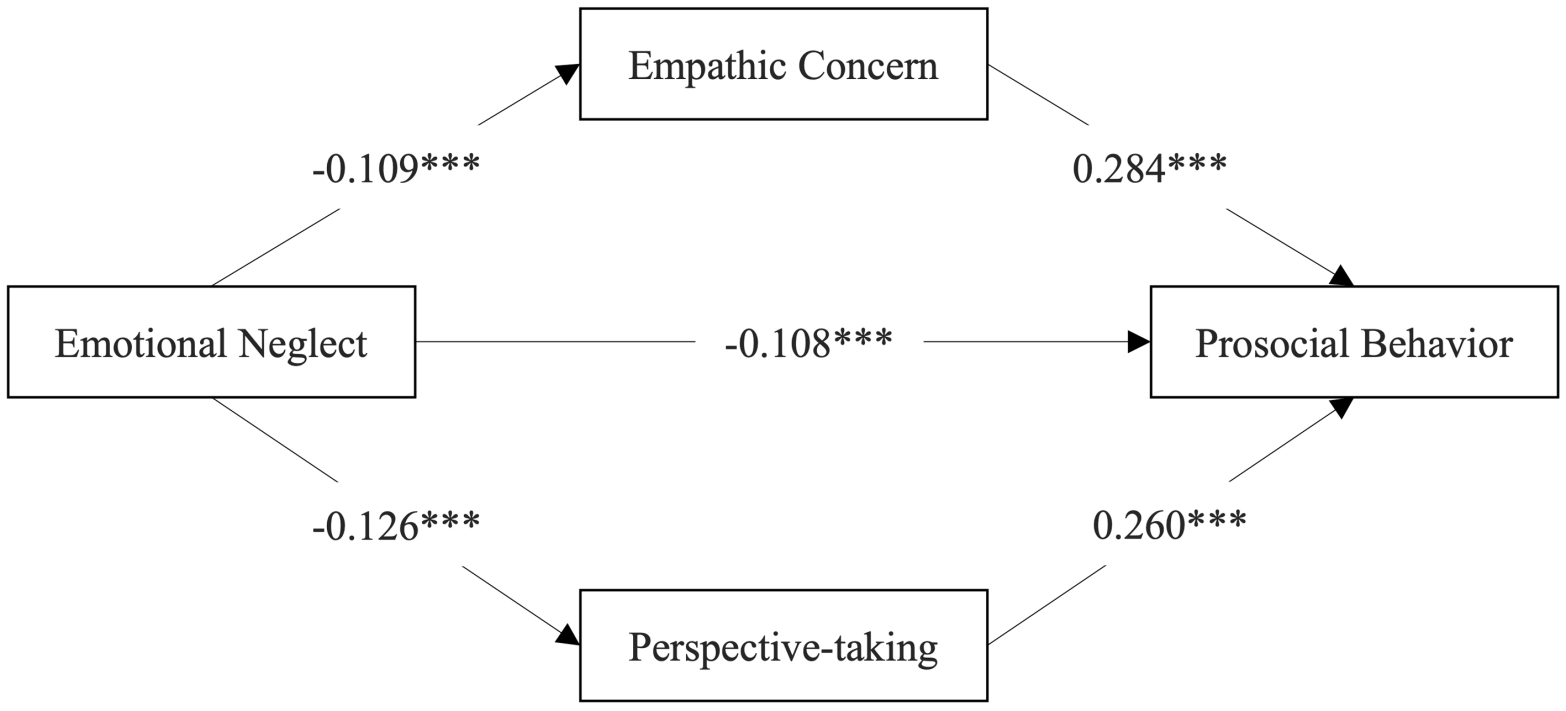
Empathic concern and perspective-taking mediate the association between emotional neglect and prosocial behavior. ***p <0.001.

After controlling for age, sex, siblings, and hometown, we found that higher emotional neglect significantly predicted worse EC and PT (*β* = −0.109, *p* = 0.001; *β* = −0.126, *p* < 0.001), which then led to lower prosocial behavior (*β* = 0.284 *p* < 0.001; *β* = 0.260, *p* < 0.001; see Table 3). The analysis also revealed a significant direct effect of emotional neglect on prosocial behavior (*β* = −0.108, *p* < 0.001; see Table 3), which indicated EC and PT were partial mediators (Figure 2). But we did not find the same mediating pathway between physical neglect and prosocial behavior. Additionally, we also did not find significant indirect effects of emotional or physical neglect on prosocial behavior through PD or FS (see Tables 3, 4).

**Table 3:** Standardized coefficient estimates predicting empathic concern, perspective-taking, personal distress, fantasy, and prosocial behavior (*N* = 1,569).

Variables	EC		PT		PD		FS		PB	
	*β* (SE)	Value of *p*	*β* (SE)	Value of *p*	*β* (SE)	Value of *p*	*β* (SE)	Value of *p*	*β* (SE)	Value of *p*
Age	0.019 (0.108)	0.450	0.011 (0.108)	0.667	−0.009 (0.143)	0.726	−0.019 (0.149)	0.439	−0.008 (0.390)	0.726
Sex	**0.077 (0.141)**	**0.003**	**0.051 (0.141)**	**0.044**	**0.180 (0.188)**	**<0.001**	**−0.111 (0.195)**	**<0.001**	0.020 (0.527)	0.387
Hometown	0.043 (0.090)	0.120	−0.007 (0.090)	0.781	**0.059 (0.120)**	**0.028**	**−0.068 (0.196)**	**0.009**	0.004 (0.328)	0.884
Siblings	0.027 (0.069)	0.333	0.043 (0.068)	0.123	−0.003 (0.091)	0.904	−0.021 (0.095)	0.439	−0.001 (0.248)	0.971
EA	0.044 (0.034)	0.091	−0.019 (0.034)	0.507	**0.167 (0.045)**	**<0.001**	**0.212 (0.046)**	**<0.001**	−0.018 (0.124)	0.483
EN	**−0.109 (0.025)**	**0.001**	**−0.126 (0.025)**	**<0.001**	0.010 (0.033)	0.758	−0.039 (0.020)	0.236	**−0.108 (0.089)**	**<0.001**
PN	−0.049 (0.040)	0.119	0.003 (0.040)	0.930	0.015 (0.054)	0.629	−0.031 (0.056)	0.319	0.005 (0.146)	0.854
EC									**0.284 (0.096)**	**<0.001**
PT									**0.260 (0.094)**	**<0.001**
PD									**−0.102 (0.071)**	**<0.001**
FS									0.043 (0.069)	0.071
*R* ^2^	**0.025**	**<0.001**	**0.021**	**<0.001**	**0.077**	**<0.001**	**0.054**	**<0.001**	**0.221**	**<0.001**
*F*	5.82		4.67		18.45		12.69		40.23	

Abuse, childhood abuse; Neglect, childhood neglect; EA, emotional abuse; EN, emotional neglect; PN, physical neglect; EC, empathic concern according to the Interpersonal Reactivity Index (IRI); PT, perspective-taking according to the IRI; PD, personal distress according to the IRI; FS, fantasy according to the IRI; PB, prosocial behavior according to the Prosocial Tendencies Measure; SE, standard error. The bold format is meant to highlight the significant values.

**Table 4:** The paths and effect analysis between childhood abuse, neglect, and prosocial behavior (*N* = 1,569).

	Effect	Path	Effect size		
			Effect	SE	95% CI
Emotional abuse model	Direct effect	Emotional abuse → prosocial behavior	−0.087	0.124	(−0.331, 0.157)
	Indirect effect	Emotional abuse → empathic concern → prosocial behavior	0.012	0.008	(−0.003, 0.028)
		Emotional abuse → perspective-taking → prosocial behavior	−0.005	0.008	(−0.020, 0.010)
		**Emotional abuse → personal distress → prosocial behavior**	**−0.017**	**0.005**	**(−0.029, −0.008)**
		Emotional abuse → fantasy → prosocial behavior	0.009	0.015	(−0.002, 0.021)
	Total effect		−0.089	0.135	(−0.353, 0.175)
Emotional neglect model	Direct effect	**Emotional neglect → prosocial behavior**	**−0.319**	**0.089**	**(−0.494, −0.144)**
	Indirect effect	**Emotional neglect → empathic concern → prosocial behavior**	**−0.091**	**0.031**	**(−0.155, −0.033)**
		**Emotional neglect → perspective-taking → prosocial behavior**	**−0.097**	**0.027**	**(−0.151, −0.048)**
		Emotional neglect → personal distress → prosocial behavior	−0.003	0.010	(−0.024, 0.017)
		Emotional neglect → fantasy → prosocial behavior	−0.005	0.006	(−0.019, 0.004)
	Total effect		−0.515	0.098	(−0.708, −0.322)
Physical neglect model	Direct effect	Physical neglect → prosocial behavior	0.027	0.146	**(−0.260, −0.313)**
	Indirect effect	Physical neglect → empathic concern → prosocial behavior	**−0.072**	**0.050**	**(−0.170, −0.027)**
		Physical neglect → perspective-taking → prosocial behavior	0.004	0.042	**(−0.078, 0.088)**
		Physical neglect → personal distress → prosocial behavior	−0.008	0.016	**(−0.041, 0.025)**
		Physical neglect → fantasy → prosocial behavior	−0.007	0.010	(−0.030, 0.008)
	Total effect		−0.056	0.162	(−0.374, 0.261)

95% CI, 95% bias-corrected confidence interval. The bold format is meant to highlight the significant values.

### Personal distress mediated the association between emotional abuse and prosocial behavior

Based on the results of Pearson’s correlation, we found only emotional abuse was significantly associated with prosocial behavior and some empathic components. Thus, we only performed mediating analysis to examine the special roles of empathic components in the relationship between emotional abuse and prosocial behavior.

**Figure 3:**
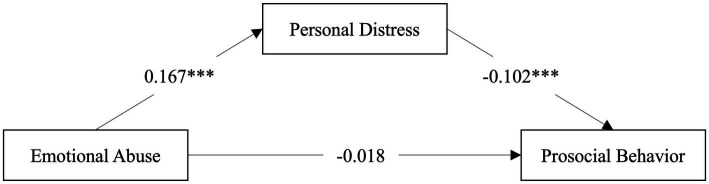
Personal distress mediates the association between emotional abuse and prosocial behavior. ***p <0.001.

After controlling for age, sex, siblings, and hometown, the mediating analysis showed that a higher level of emotional abuse significantly predicted higher PD (*β* = 0.167, *p* < 0.001), and PD negatively predicted prosocial behavior (*β* = −0.102, *p* < 0.001). However, the direct path from emotional abuse to prosocial behavior was not statistically significant (*β* = −0.018, *p* = 0.483; see Table 3). Therefore, PD completely mediated the relationship between emotional abuse and prosocial behavior (Figure 3). Moreover, EC, PT and FS did not mediate the relationship between emotional abuse and prosocial behavior (see Tables 3, 4).

## Discussion

The purpose of the current study was to investigate the distinct mediating effects of different empathic components in the relationships between different forms of childhood maltreatment and prosocial behavior. We found that emotional abuse dampened prosocial behavior by increasing PD. Emotional neglect not only reduced prosocial behavior directly, but also through the mediating pathway of lowering EC and PT. Physical abuse, sexual abuse and physical neglect have little effect on prosocial behavior. The current findings support our hypothesis that abuse and neglect impact prosocial behavior *via* distinct pathways, and prove that emotional maltreatment have more significant effect on prosocial behavior and empathic components than physical or sexual maltreatment. The present study helps us to better understand the influential mechanisms underlying the effect of abuse/neglect on prosocial behavior.

According to previous research, relative to males, females showed higher emotional responsivity and mirroring responses to others’ pain which present stronger overall emotional empathy (Christov-Moore et al., 2014). The present study found that females showed higher EC and PD than males, which was consistent with the existing results (Schulte-Rüther et al., 2008; Birkett, 2014). In contrast, few studies have explored gender difference in cognitive empathy (including FS and PT) and its underling mechanism. In the current study, males showed higher FS than females, but no significant gender difference in PT was found. The current results indicate that gender difference in different empathic components exists and is needed to be confirmed in the future studies. Stereotypically, females are portrayed as more prosocial than males. However, we did not find gender difference in prosocial behavior in college students. The present study did not find significant difference in abuse or neglect experience either.

### The mediating role of empathic concern and perspective taking in the emotional neglect model

The present findings are basically in line with Miano’s et al. (2018) research which found that neglect predicted decreased empathic accuracy (one of the manifestations of weak PT). We further found that only emotional neglect impaired PT and EC abilities, and in turn contributed to decreased prosocial behavior, while physical neglect did not. Unlike physical neglect, which refers to the failure to provide children with adequate food, clothing and medical care, and largely relys on the economic status of the family, emotional neglect manifested by parents’ refusal to interact with their children and to meet children’s emotional needs seems to have more profound effect on childrens’ social development.

There are two possible explanations for the deleterious effect of emotional neglect on PT. Firstly, parents or other caregivers are the main environmental resource for children to acquire social-cognitive ability in early life. Chronic emotional neglect deprives children of the adequate chance to develop social functioning. Second, the parents who emotionally neglect their children usually present with a deficit in processing social information (such as a failure to recognize a child’s emotional state or correctly interpret the signal of need; Crittenden, 1993), which means they are less able to provide a good template for children to learn social skills. Children reared in these environments are more likely to acquire poor social information processing skills, including low PT skills. Consistent with previous findings (Blankenstein et al., 2020), weak PT predicted lower prosocial behavior. Some scholars have suggested that the perception of others’ mental states is the basic prerequisite for arousal of an altruistic attitude and prosocial decision-making (Carlo et al., 1999; Kanske et al., 2015). Due to the inability to recognize other’s situation and emotional state, individuals who encountered emotional neglect during childhood might not possess the capacity necessary to generate prosocial tendency, let alone prosocial behavior. Our findings indicated that training of PT or the theory of mind would help to promote prosocial behavior in emotionally neglected individuals.

According to the attachment theory (Bowlby, 2008), children are unlikely to develop secure attachment styles when their attachment figures are unresponsive and unavailable (especially in parents who tends to fail to meet their children’s emotional need), and these styles extend to later personal interactions. Empirical research has demonstrated that emotional neglect causes insecure attachment in adulthood, which mainly presents as attachment avoidance or attachment anxiety (Huh et al., 2020; Struck et al., 2020). Additionally, weak attachment has been found to strongly decrease the magnitude of EC (Batson and Shaw, 1991). According to the prosocial motivation theory, EC is the core empathic component that drives altruistic motivation and promotes prosocial behavior (Batson and Shaw, 1991). Our findings that low EC predicted fewer prosocial behavior is in lined with the prosocial motivation theory, as well as previous empirical studies (Carlo et al., 2015; Kamas and Preston, 2021). Emotionally neglected individuals who are unable to establish solid relationships with others and cannot understand the feelings of those in need, are unlikely to engage in prosocial behavior, even if they can correctly adopt the perspective of others. Therefore, training social interaction and compassion could help to promote prosocial behavior in neglected individuals.

What’s more, FS and PD did not mediate the relationship between emotional or physical neglect and prosocial behavior. Previous findings have found that FS and PD showed large differences in different populations. For example, the American population has been found to have higher FS than people in other countries (Birkett, 2014), and depressive people showed more PD than the general population (Zhang et al., 2021). To our knowledge, ours is the first study to examine the relationships between neglect and FS and PD. Therefore, the results based on Chinese young adults should be interpreted with caution; more investigations with other populations are needed to verify these results and explore the potential mechanisms underlying these relationships in more depth.

### The mediating role of personal distress in the emotional abuse model

We found that emotional abuse increased PD, which in turn inhibited prosocial behavior, while physical abuse and sexual abuse showed little effect on prosocial behavior and empathy, which might suggest that emotional abuse causes more significant disruption on social emotions and behaviors than other forms of abuse. EC and PD are two different kinds of oriented emotional empathy. Decety (2011) argued that emotional regulation plays an important role in determining the orientation of emotional empathy (i.e., whether it develops into PD or EC). Individuals who suffer from abuse have been found to have poor emotional regulation ability, as indicated by reduced activity in control-related brain regions (Blair et al., 2019). Ineffective emotional regulation makes individuals who have been emotionally abused prone to high levels of PD in the face of others’ bad situations, and this unease and disturbance might not benefit helping behavior, but even inhibit it (Davis, 1983; Eisenberg and Eggum, 2009). The current study revealed that a high level of PD predicted less prosocial behavior. It is believed that a high level of PD evokes egoistic motivation that acts to reduce aversive arousal, rather than altruistic motivation to help needy individuals (Batson, 1987). To summarize, the over-arousal of negative emotions caused by emotional abuse reduces the willingness to engage in prosocial behavior.

Unlike emotional neglect, we found no significant effects of emotional abuse on PT or EC. Compared to individuals who have suffered from emotional neglect, those who have been emotionally abused appear to preserve a relatively normal empathic function. These findings were in line with some existing studies. For example, Miano et al. (2018) suggested that overall abuse did not decrease cognitive empathy and Ometto et al. (2016) showed that physical abuse did not affect compassion, but this study did not examine the effect of emotional abuse. However, a study whose participants aged from 4 to 10 years old found that abuse decreased children’s cognitive empathy but did not decrease their emotional empathy (Meidan and Uzefovsky, 2020), which was not exactly consistent with the current finding. We suggest that it is necessary to further examine the relationships between different forms of abuse and PT and EC in more larger populations of different age groups.

Moreover, we found that emotional abuse could significantly increase FS, but FS was unrelated to prosocial behavior. Daydreaming or FS has been found to benefit the regulation of mental distress caused by childhood abuse and neglect (Somer et al., 2021). The current study found that only emotional abuse predicted high level of general FS, and other forms of maltreatment did not. Moreover, previous results on the effect of FS on prosocial behavior have been mixed (Tahiroglu and Taylor, 2019; Pang et al., 2022). We proposed that distinguishing different elements of FS would help to provide a deeper insight into the special role of FS in the association between childhood maltreatment and prosocial behavior.

### Emotional abuse/neglect and prosocial behavior

The current study showed that only emotional neglect had a direct impact on prosocial behavior, in Chinese young adults, which was consistent with Gomis-Pomares and Villanueva (2020) based on Spanish population. This suggests that the unique effect of emotional neglect on prosocial behavior is consistent across populations. In addition to the deficits of PT and EC, there exist some other factors in individuals who have been neglected in emotional need that could affect prosocial behavior, such as caring capacity, subjective willingness to help, and the social reward circuit (Fehr and Rockenbach, 2003; Grueneisen and Warneken, 2022). Individuals who have never felt loved or important might be unable to acquire the ability to care or love others. Furthermore, these deficits may cause more social withdrawal and limited interpersonal relationships, which further decrease interest in caring for others (Music, 2011). Additionally, some studies have found that neglect predicts the hyposensitivity of reward and blunted reward processing (Bounoua et al., 2021; Yang et al., 2021). In this case, children who have experienced neglect would be less likely to associate prosocial behavior with positive social consequences (such as obtaining social reward or a good reputation or avoiding social punishment), which would in turn reduce the willingness to engage in prosocial behavior.

We found no significant direct path from emotional abuse to prosocial behavior, which indicates that PD completely mediated the relationship between emotional abuse and prosocial behavior. This indicates that emotional abuse only reduced the prosocial behavior *via* over-arousal of distress.

Abuse has received an increasing amount of attention in recent decades, largely because it can cause obvious damage in a short period of time and has a stronger impact on psychiatric disorders and externalizing behavior than neglect does (Liu et al., 2018; Strathearn et al., 2020). Unfortunately, the effects of childhood neglect are often neglected, despite the fact that far more children are neglected than abused, especially in China. According to national statistics, there were around 6.97 million left-behind children (neglected children) in 2018 (Ge et al., 2022). What’s more, emotional neglect is more common now, because some parents believe that adequate food and safe environment are enough for children’ development. Considering the chronic and profound effect of emotional neglect on social functioning, future studies should focus on this. The current study provides a comprehensive understanding on how abuse/neglect affect prosocial behavior through different empathic components and might help to establish targeted psychological interventions to improve prosocial behavior in individuals who have been maltreated. Specifically, we suggest that trainings of empathy (that are targeted to promote EC and PT) and social interaction would be suitable for individuals who have been emotionally neglected, and training of emotional regulation could be useful for individuals who have been emotionally abused.

## Limitations

Our study has several limitations that should be noted. First, due to the nature of cross-sectional studies, it is not possible for us to infer causality in the relationships between childhood abuse/neglect, different empathic components, and prosocial behavior. Further longitudinal studies or randomized controlled intervention experiments are needed to examine the causal relationships between these variables. Second, the present study totally depended on self-report measures. Although we adopted the well-established questionnaires, like the Childhood Trauma Questionnaire-Short Form, which has been confirmed to have a low false-positive rate and (Teicher et al., 2016), it could be useful to combine with data obtained from interviews with both children and their parents. This would help us to better understand the outcomes of childhood maltreatment. Third, we only explored several separate forms of early-life maltreatment in the current study. Other aspects of maltreatment that were excluded in our research, such as low socioeconomic status or school bullying, might exert different effects on prosocial behavior. Fourth, most of the effect sizes in the present results were small according to the criterion proposed by Sullivan and Feinn (2012). We speculated that the main reason for the relatively small effects could be attributed to the characteristics of college students in the current study. Compared to the orphans in welfare institution and the abused children reported by the government (Teicher et al., 2016), college students have relatively mild abused or neglected experience. The effect sizes between maltreatment experiences and prosocial behavior might be stronger in the sample such as rescue stations, shelters, and foster care who suffered from more serious abuse and neglect. Future research could compare and supplement the current results by collecting multicenter data.

## Conclusion

This study revealed that childhood abuse and neglect have differential effects on prosocial behavior. To be specific, PT and EC played partially mediating roles in the association between emotional neglect and prosocial behavior. PD completely mediated the relationship between emotional abuse and prosocial behavior. Physical abuse, sexual abuse and physical neglect have little effect on prosocial behavior. The present findings offer a better understanding of how abuse and neglect differently affect prosocial behavior through different empathic components, and provide a platform for future directions, such as the development of targeted psychological interventions for different types of maltreatment.

## Data availability statement

The raw data supporting the conclusions of this article will be made available by the authors, without undue reservation.

## Ethics statement

The studies involving human participants were reviewed and approved by the Ethics Committee of Southern Medical University. The participants provided their written informed consent to participate in this study. Written informed consent was obtained from a legal guardian/next of kin of participants under the age of 18.

## Author contributions

YW and XuY designed the research. PC, QZ, XS, and XiY performed the research and analyzed data. PC and QZ wrote the manuscript. YW and XuY critically reviewed the manuscript. All authors contributed to the article and approved the submitted version.

## Funding

This study was supported by the National Natural Science Foundation of China (grant number: 31800928); Humanities and Social Sciences Youth Project of the Ministry of Education, China (grant number: 22YJCZH182).

## Conflict of interest

The authors declare that the research was conducted in the absence of any commercial or financial relationships that could be construed as a potential conflict of interest.

## Publisher’s note

All claims expressed in this article are solely those of the authors and do not necessarily represent those of their affiliated organizations, or those of the publisher, the editors and the reviewers. Any product that may be evaluated in this article, or claim that may be made by its manufacturer, is not guaranteed or endorsed by the publisher.
